# Exploring the energy landscape of a SAM-I riboswitch

**DOI:** 10.1007/s10867-021-09584-7

**Published:** 2021-10-26

**Authors:** Christoph Manz, Andrei Yu Kobitski, Ayan Samanta, Karin Nienhaus, Andres Jäschke, Gerd Ulrich Nienhaus

**Affiliations:** 1 Institute of Applied Physics, Karlsruhe Institute of Technology, Wolfgang-Gaede-Str. 1, 76131 Karlsruhe, Germany; 2 Institute of Pharmacy and Molecular Biotechnology, Heidelberg University, Im Neuenheimer Feld 364, 69120 Heidelberg, Germany; 3 Present Address: Department of Chemistry, Uppsala University, Box 538, 751 21 Uppsala, Sweden; 4 Institute of Nanotechnology, Karlsruhe Institute of Technology, Hermann-von-Helmholtz-Platz 1, 76344 Eggenstein-Leopoldshafen, Germany; 5 Institute of Biological and Chemical Systems, Karlsruhe Institute of Technology, Hermann-von-Helmholtz-Platz 1, 76344 Eggenstein-Leopoldshafen, Germany; 6 Department of Physics, University of Illinois at Urbana-Champaign, 1110 W. Green St, Urbana, IL 61801 USA

**Keywords:** Sam-I riboswitch, Förster Resonance Energy Transfer (FRET), Hidden Markov model (HMM), Energy landscape

## Abstract

SAM-I riboswitches regulate gene expression through transcription termination upon binding a *S*-adenosyl-L-methionine (SAM) ligand. In previous work, we characterized the conformational energy landscape of the full-length *Bacillus subtilis yitJ* SAM-I riboswitch as a function of Mg^2+^ and SAM ligand concentrations. Here, we have extended this work with measurements on a structurally similar ligand, *S*-adenosyl-l-homocysteine (SAH), which has, however, a much lower binding affinity. Using single-molecule Förster resonance energy transfer (smFRET) microscopy and hidden Markov modeling (HMM) analysis, we identified major conformations and determined their fractional populations and dynamics. At high Mg^2+^ concentration, FRET analysis yielded four distinct conformations, which we assigned to two terminator and two antiterminator states. In the same solvent, but with SAM added at saturating concentrations, four states persisted, although their populations, lifetimes and interconversion dynamics changed. In the presence of SAH instead of SAM, HMM revealed again four well-populated states and, in addition, a weakly populated ‘hub’ state that appears to mediate conformational transitions between three of the other states. Our data show pronounced and specific effects of the SAM and SAH ligands on the RNA conformational energy landscape. Interestingly, both SAM and SAH shifted the fractional populations toward terminator folds, but only gradually, so the effect cannot explain the switching action. Instead, we propose that the noticeably accelerated dynamics of interconversion between terminator and antiterminator states upon SAM binding may be essential for control of transcription.

## Introduction

**Fig. 1:**
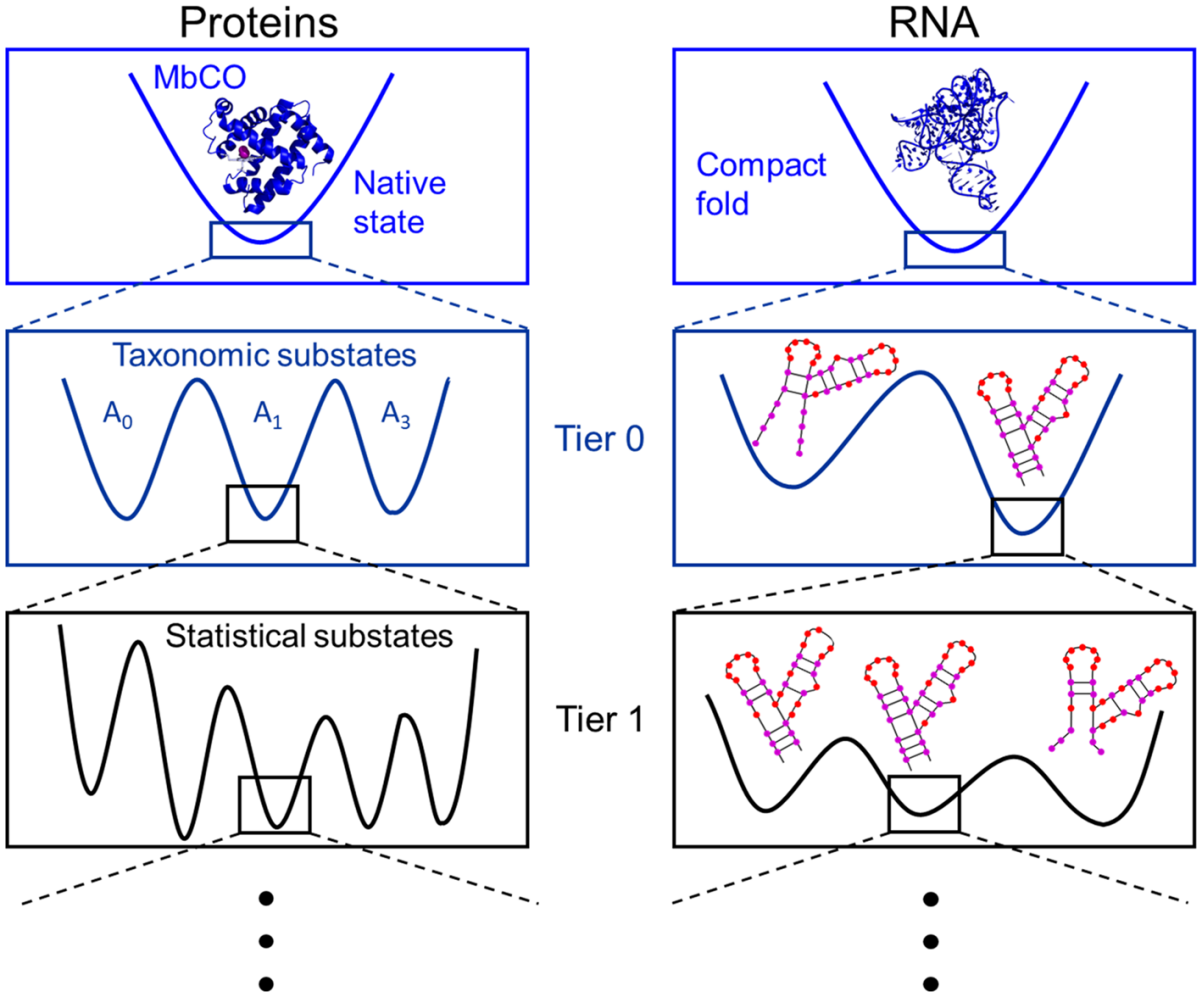
Conformational free energy landscapes of biopolymers. Proteins and RNA molecules are dynamic systems and fluctuate between many different conformations. The energy landscape is arranged hierarchically, with conformations nested in several tiers, according to the barriers separating the states

The energy landscape is an overarching concept characterizing systems as diverse as glasses, spin glasses, synthetic and biological polymers, evolution, immunology and neural networks [1, 2]. Hans Frauenfelder, whose upcoming 100th birthday is the occasion of this paper, pioneered the use of energy landscapes for the description of the structural heterogeneity of proteins and its connection to functional processes. Proteins display a vast number of conformational substates that can be represented by minima in a conformational energy landscape, specifying the energy of every possible configuration of atoms. In 1985, Frauenfelder and coworkers published a seminal paper, in which they integrated a large body of experimental data taken on myoglobin, ‘the hydrogen atom of biology’ [3], to propose a hierarchical arrangement of conformational substates (Fig. 1) [4]. (Interestingly, the notion of an energy landscape—in this context—was only coined some years later [5]). In carbonmonoxy myoglobin, a few taxonomic substates exist on the highest level, denoted Tier 0. They correspond to energy minima separated by high free-energy barriers and can be characterized individually in terms of their structural, spectroscopic and energetic properties [2, 6–9]. Each taxonomic substate harbors a large number of statistical substates of lower tiers (1, 2, 3), separated by successively smaller energy barriers. Protein relaxations in response to perturbations as well as equilibrium fluctuations are governed by the specific shape of the energy landscape [4]. Environmental cues (e.g., ligand binding, solvent properties) can sculpt the energy landscape to enable proteins to perform specific functions [2, 10].

Like proteins, nucleic acids are linear heteropolymers endowed with a huge number of conformational degrees of freedom. Notably, ribonucleic acid (RNA) molecules can fold into fascinating densely packed three-dimensional architectures, and play key enzymatic roles in protein biosynthesis and other processes. Not surprisingly, the energy landscape paradigm has been adopted as a useful general framework for RNA molecules [11, 12], describing their structural heterogeneity and (temporally widely dispersed) dynamics. Due to the predominant contribution of hydrogen bonds formed upon base pairing to the stabilization of nucleic acids, which typically results in a few distinct secondary structure patterns for small RNAs, the energy landscape is strongly hierarchical (Fig. 1). Borrowing from Frauenfelder et al. [5], Mustoe et al. [12] recently assigned RNA structural properties and dynamics to different tiers of a hierarchical free energy landscape. In Tier 0, there are typically a few conformations with distinct secondary structure patterns. Interconversions between Tier 0 states are the slowest and take more than 100 ms (at room temperature). Within each of them, there are multiple Tier 1 states, separated by smaller barriers. They display small differences in base pairing or tertiary contacts. Tier 1 states are again split into Tier 2 conformations, characterized by different arrangements of helices, loops and local structures. As for proteins, environmental cues may modify the energy landscape to control functional processes. For example, selective stabilization of a particular Tier 0 state, e.g., due to ligand binding, may switch the RNA molecule to a very different yet well-defined fold.

**Fig. 2:**
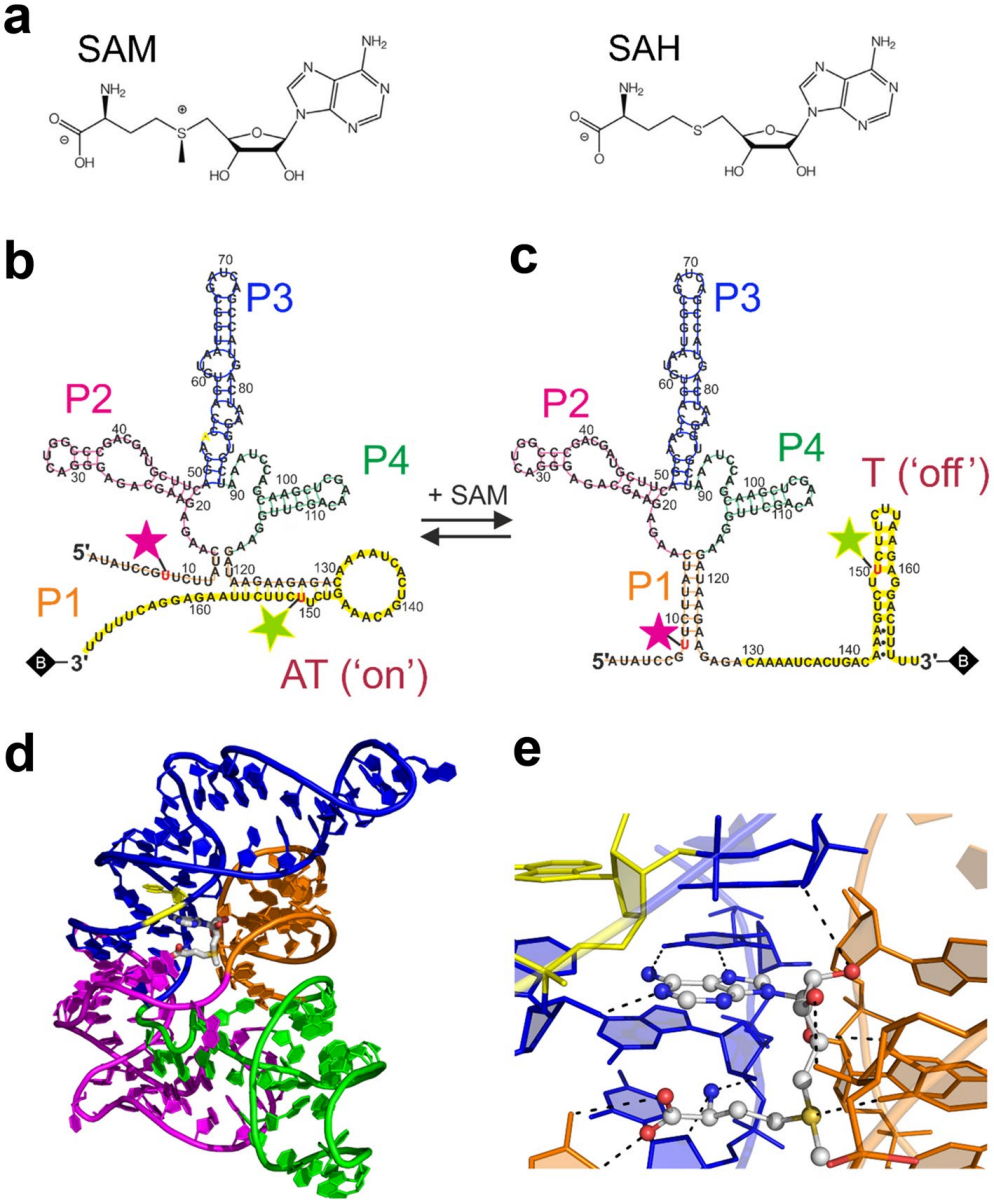
Structural depictions of the SAM-I riboswitch and ligands. **a** Chemical structures of the SAM and SAH metabolites. **b**, **c** Secondary structure models of the *B. subtilis yitJ* SAM-I riboswitch (169-nt fragment) in the suggested **b** antiterminator (AT) and **c** terminator (T) conformations. Helices P1 (orange), P2 (magenta), P3 (blue) and P4 (green) form the aptamer; the expression platform is colored in yellow. Base pairing of the T structure and the AT stem loop is depicted by lines according to previous work [17]. Donor (Cy3) and acceptor (Cy5) dyes attached to uridines (in red letters) of the construct are shown as green and red stars, respectively. Biotin is represented by a black diamond at the 3ʹ-end. **d** Cartoon representation of the 3D SAM-I riboswitch aptamer structure (PDB ID 4KQY [15]). Helices are color-coded as in **b**. SAM is depicted in stick representation, with carbons plotted in white, oxygen in red, nitrogen in blue and sulfur in yellow. **e** Close-up of the SAM ligand, with the atoms depicted as small spheres (color coding as in **d**). Dotted lines: hydrogen bonding interactions. Presumably, the adenosyl moiety of SAM has displaced A54 (in yellow), as was reported for the SAM-I riboswitch from *Thermoanaerobacter tengcongensis* [21]

As an example of RNA free energy landscape exploration, we present here our recent single molecule Förster resonance energy transfer (smFRET) studies of a bacterial riboswitch. Riboswitches are highly structured, noncoding RNA elements that can be found in the 5’ untranslated regions of some bacterial messenger RNAs. Oftentimes, they specifically recognize important metabolites, and exert control over the expression of enzymes involved in their synthesis, which are encoded downstream of the riboswitch sequence on the messenger RNA. This gene regulation can occur on the transcriptional or the translational level. To give an example, the SAM-I riboswitch senses the presence of *S*-adenosyl-l-methionine (SAM, Fig. 2a) and modulates gene transcription in response. It includes two domains, the aptamer domain and the expression platform. The aptamer serves as a biosensor, capturing the target metabolite SAM in a ligand-binding pocket with high specificity and selectivity. Its sequence partially overlaps with that of the expression platform, which, in the simplest model, toggles between two distinct secondary structures, denoted terminator (T) and antiterminator (AT), depending on whether a SAM ligand is bound or not, respectively (Fig. 2b, c). The AT stem-loop structure (Fig. 2b) leads to transcriptional read-through (ON state), whereas formation of a rho-independent (intrinsic) T stem-loop (Fig. 2c) results in premature transcription termination (OFF state).

To probe conformational heterogeneity and dynamics of the SAM-I riboswitch using smFRET, we attached a donor and an acceptor dye to selected uridine nucleotides on the RNA chain (see Fig. 2b, c). Their positions need to be suitably chosen, so that conformational transitions result in noticeable FRET efficiency changes, which depend on the inverse sixth power of the inter-dye distance. To analyze the presence of multiple conformations in equilibrium via FRET, the simplest approach is to use a confocal microscope to excite the donor and then register photon emission from the donor and acceptor, while riboswitch molecules diffuse through the observation volume of ca. 1 fl (1 µm^3^) in an aqueous solution. From the photon emission from thousands of molecules, FRET efficiency values are calculated and compiled in histograms displaying the number of molecules within certain FRET value intervals. If a small number of discrete conformations exists that are well separated in the FRET histogram, they can be disentangled by fitting multiple Gaussians. Despite the simple, two-state mechanistic description of riboswitch action sketched above, FRET histogram analysis of the riboswitch yielded broad, not well resolvable distributions. Thus, we measured the fluorescence emission from individual immobilized riboswitch molecules as a function of time, and we applied Hidden Markov Model (HMM) analysis to our smFRET data. Unlike FRET efficiency histogram analysis, which relies solely on spectroscopic information, HMM exploits both spectroscopic and temporal information by assuming stochastic switching between a set of discrete states with well-defined average FRET efficiencies and state lifetimes, and provides rate coefficients of conformational transitions on timescales from submilliseconds to seconds. Therefore, smFRET analysis yields equilibrium and kinetic properties of major (Tier 0) conformations. By comparing results on the SAM-I riboswitch without a ligand to those in the presence of SAM and S-adenosyl-L-homocysteine (SAH), we observed how the free energy landscape is modified by ligand binding and draw conclusions for the biological function of the SAM-I riboswitch.

## SAM-I riboswitch

For our study, we have investigated the 169-nucleotide SAM-I riboswitch from the *Bacillus subtilis yitJ* gene, which regulates transcription of an enzyme involved in methionine synthesis. SAM is an important metabolite, acting as a methyl group donor in many biochemical reactions. It contains a positively charged sulfonium center substituted by the methyl group that is being transferred (Fig. 2a). In the demethylation reaction, SAM is converted to the neutral compound, SAH (Fig. 2a), which is toxic for the cell at high concentrations and rapidly degraded by SAH-nucleosidases or SAH-hydrolases [13]. The SAM-I riboswitch is highly selective for SAM; it binds SAM with an equilibrium dissociation coefficient, *K*_D_, of 19 nM [14], which is two orders of magnitude smaller than the one for SAH [15, 16].

Figure 2b and c shows secondary structures of the SAM-I riboswitch in the ON (AT) and OFF (T) states, as suggested by previous publications [17–19], based on chemical probing experiments [20]. Direct structural information by X-ray crystallography is only available for the compact aptamer domain (with bound SAM ligand, PDB code 4KQY) [15, 21]. The aptamer domain consists of four helices (P1–P4; Fig. 2) that are joined together by a four-way junction. A compact tertiary fold featuring a kink-turn (KT) motif in helix P2 as well as a pseudoknot (PK) motif involving P2 and the P3–P4 joining region creates a pocket formed by helices P1 and P3 and the P1–P2 joining region in which the SAM ligand binds in a U-shaped fashion (Fig. 2d, e). Helices P2–P4 are consecutive hairpin structures, whereas helix P1 forms by long-range pairing of strand segments from the ends of the aptamer sequence. SAM ligand binding stabilizes this helix, which appears to be important for the switching function of the SAM riboswitch and other P1-helix-regulated riboswitches [22]. Notably, an alternative secondary fold has been proposed for the AT form of the SAM-I riboswitch from *Thermoanaerobacter tengcongensis* [23], although the crystal structure of its aptamer domain (with SAM, PDB code 3IQP [21]) is practically identical to the one of *Bacillus subtilis yitJ*. The overall structure of the aptamer domain without SAM (PDB code 3IQR [21]) is identical to the SAM-bound one, with the exception that a RNA adenosyl moiety occupies the site of the SAM adenosyl (Fig. 2e). Unfortunately, only a few papers have addressed the interplay between the aptamer and expression platform so far [24–26], and a crystal structure of the full-length riboswitch has not yet become available.

We synthesized the full-length riboswitch from five RNA oligomers by splinted ligation [27]. A biotin moiety was attached at the 3’-end for immobilization on a glass surface via streptavidin. For FRET dye attachment, we examined a number of sites based on prior knowledge of the secondary and tertiary structures [15, 21]. The work shown here was performed with a riboswitch construct that has the donor located on the expression platform (U150) and the acceptor on the P1 helix of the aptamer domain (U8), and thus senses conformational changes of the expression platform with respect to the aptamer domain (Fig. 2b).

## Single-molecule FRET measurements and analysis

The experiments were performed on a home-built confocal microscope based on a Zeiss Axiovert 35 frame, modified from a previously described design [28, 29]. Most importantly, we included a programmable beam splitter to achieve high detection efficiency in multicolor excitation and detection [30]. Cy3 (donor) and Cy5 (acceptor) dyes were excited by a 532-nm laser (Excelsior 532, Nd:YAG laser; Spectra Physics, Mountain View, CA) and a 637-nm laser (Obis 637; Coherent, Santa Clara, CA), respectively. We employed an alternating laser excitation (ALEX) scheme [31] by continually switching between donor (70 µs) and acceptor (30 µs) excitation, with a 5 µs blank interval in the detection during switching to avoid temporal crosstalk. The fluorescence emission was collected by a water immersion objective (UPlan Apo 60 × /1.2w; Olympus, Hamburg, Germany), passed through a pinhole (diameter: 100 μm for freely diffusing molecules, 50 μm for surface-immobilized molecules), and separated by a dichroic mirror (640DCXR; Chroma, Bellows Falls, VT) into donor (green) and acceptor (red) color channels. After passing through filters (BrightLine HC 580/60 and HC 642/LP for the green and red channels, respectively; Semrock, Rochester, NY), single photons were detected by avalanche photodiodes (APD; SPCM-AQR-14; PerkinElmer Optoelectronics, Boston, MA). Counts were registered by a data acquisition card (PCI 6602; National Instruments) synchronized with the ALEX cycle. A XY piezoelectric stage (P-731.20; Physik Instrumente, Karlsruhe, Germany) with analog voltage control by a multifunctional data acquisition card (PCI 6229; National Instruments, Austin, TX) was used to position the samples. We acquired data on immobilized molecules by moving the piezo stage across fields of 30 × 30 μm^2^ to collect images of 128 × 128 pixels with 5 ms pixel dwell time. Samples of freely diffusing molecules were continuously moved along a circle with a 30-μm diameter at 25 μm s^−1^ to avoid optically biased diffusion effects. A program written in C++ allowed for real-time control of all electronic devices and automatic data acquisition.

For FRET histogram analysis, SAM-I riboswitch molecules were dissolved at 50 pM in 50 mM Tris, 100 mM NaCl, pH 7.4, supplemented with the desired concentrations of Mg^2+^ ions and SAM or SAH ligands. Several thousand photon bursts from single molecules diffusing through the focus were collected to calculate FRET efficiency values by ratiometric analysis of donor and acceptor intensities.

Donor and acceptor intensity time traces on immobilized molecules were collected on samples kept in home-made microfluidic chambers constructed from two glass coverslips. For immobilization, the sample chamber was incubated with a streptavidin solution (10 μg/ml in phosphate-buffered saline) to adhere the protein to the glass surface. Then, the sample solution was added so that the riboswitch molecules bound to streptavidin via their biotin moiety at the 3’ end. The Mg^2+^ concentration was 15 mM, and the SAM and SAH concentrations were 10 µM, respectively. Dye photobleaching was minimized by adding oxygen scavenging [32] and triplet quenching [33] systems (1 μM protocatechuate 3,4-dioxygenase, 2 mM protocatechuic acid, 1 mM Trolox, 1 mM cyclooctatetraene, 1 mM nitrobenzyl alcohol, and 1 mM 2-mercaptoethylamine) to the sample solution immediately before the measurement. To observe interconversion between SAM-I riboswitch conformations, we recorded donor and acceptor intensity time traces of a large number of individual, immobilized RNA molecules. Only those traces showing anti-correlated donor and acceptor intensity fluctuations and single-step photobleaching after an extended time period were converted into FRET efficiency time traces using the equation *E* = *I*_A_/(*I*_A_ + *γI*_D_). Here, *I*_A_ and *I*_D_ are the (background-corrected) photon counts in the acceptor and donor channels, respectively, and *γ* is a correction factor accounting for different dye quantum yields and photon detection efficiencies in the two color channels.

The donor and acceptor intensity time traces were subsequently submitted to HMM analysis. HMM is a sophisticated algorithm that overcomes the serious problem of overlapping FRET distributions, which prevents unambiguous assignment of a measured FRET value to a particular conformation. HMM invokes a kinetic scheme comprising a set of interconverting states with exponential lifetime distributions. A likelihood function is constructed that assesses how well a pre-defined HMM matches the observed FRET data. Notably, the number of states needs to be thoroughly assessed (and kept to a minimum) by the user. The choice of the proper HMM scheme is a key step that requires thorough validation, ensuring that the data are indeed consistent with Markovian state dynamics. Thus, by using HMM analysis, different conformations are not only distinguished by their FRET efficiency distributions, but also by their interconversion kinetics and, thus, state lifetimes. A detailed algorithm of smFRET HMM analysis, including optimization and validation procedures, is described in [34].

## Mg^2+^ and ligand concentration dependence of smFRET histograms

**Fig. 3:**
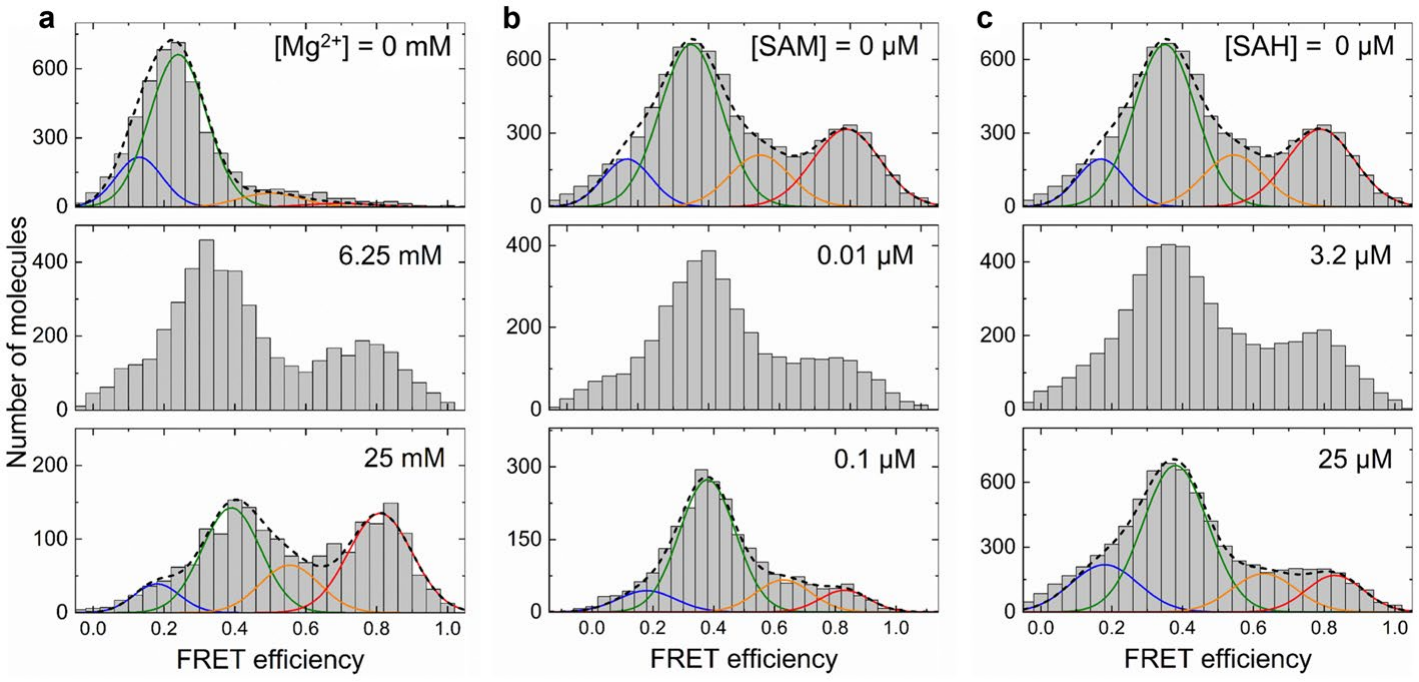
Histograms of smFRET efficiency values of freely diffusing SAM-I riboswitch constructs exposed to different buffer solutions. **a** 0, 6.25 and 25 mM Mg^2+^. **b** 0 nM, 10 nM and 100 nM SAM in the presence of 20 mM Mg^2+^. **c** 0 nM, 3.2 µM and 25 µM SAH in the presence of 20 mM Mg^2+^. Lines: best-fit Gaussian distributions of four individual subconformations (colored lines) and their sums (dashed lines)

To study the effect of varying Mg^2+^ concentration as well as the presence of SAM or SAH ligands on the FRET efficiencies, FRET efficiency values of thousands of molecules were calculated and compiled in histograms (Fig. 3). The resulting distributions are broad and cover essentially the entire range between 0 and 1. To extract discrete subpopulations, presumably Tier 0 conformations, it is customary in the field to fit multiple Gaussians to the histograms (colored lines in Fig. 3). Usually, histogram data at a single concentration do not suffice to disentangle the FRET distributions due to overlap. In our smFRET studies of RNA molecules, we thus collect many histograms with varying Mg^2+^ or ligand concentrations and perform global fits with discrete sets of subdistributions [29, 35, 36].

Figure 3a shows histograms of smFRET efficiency values of the SAM-I riboswitch construct exposed to buffer solutions at three different Mg^2+^ concentrations, 0, 6.25 and 25 mM. In the absence of Mg^2+^, most molecules adopt a low-FRET conformation with an average FRET efficiency, 〈*E*〉 ≈ 0.2, which corresponds to an inter-dye distance of ~ 67 Å. We modeled the distribution with two Gaussians (Fig. 3a, blue and green). According to Hennely et al. [24], the aptamer domain of the riboswitch preferentially adopts the T conformation at low Mg^2+^ concentration. The FRET peak shifts to higher FRET efficiency with increasing Mg^2+^ concentration, indicating that the riboswitch acquires a more compact structure. Moreover, a high-FRET population with *E* > 0.6 (Fig. 3a, modeled with two Gaussians depicted in orange and red) emerges at the expense of the low-FRET population, indicating that the expression platform moiety associates more closely with the aptamer. This compact tertiary structure has been assigned to an AT conformation, in which the AT hairpin is stacked onto a partially formed P1 helix [25]. At 25 mM Mg^2+^, high-FRET and low-FRET states are populated roughly evenly. In agreement with our findings, Roy et al. [37] recently reported that the aptamer domain undergoes a collapse transition (with a midpoint concentration, [Mg^2+^]_0.5_, of 0.4 mM Mg^2+^) at low Mg^2+^ concentrations and pre-organizes into an OFF state (T), whereas the ON state (AT) became populated at higher Mg^2+^ concentrations.

Upon addition of increasing amounts of SAM to the riboswitch in buffer solution supplemented with 20 mM Mg^2+^ (to ensure a substantial fraction of high-FRET molecules), the equilibrium population of conformations shifted back to low-FRET states (Fig. 3b). This finding supports the assignment of the high-FRET population to AT conformations, as SAM binding is known to stabilize T conformations, which have greater inter-dye distances due to T hairpin formation [16]. Based on X-ray structure analyses of the aptamer domain of the *Thermoanaerobacter tengcongensis* SAM-I riboswitch, Montage et al. [38] reported that van der Waals interactions serve as the principal means of orienting the ligand in the binding pocket. Moreover, the selectivity toward SAM with respect to similar ligands such as SAH derives from electrostatic interactions between the SAM sulfonium ion and uracil carbonyls located in the minor groove of the P1 helix, stabilizing the P1 helix [23]. As a result, the T conformation is favored. We note that the SAM-induced shift from AT to T conformations is rather limited at high Mg^2+^; without SAM, ca. 40% of the riboswitch molecules (orange and red Gaussians in Fig. 3b) are in an AT conformation; at 100 nM SAM, this value is decreased to 25%.

FRET histograms at three SAH concentrations are depicted in Fig. 3c. There is a population shift from high-FRET to low-FRET conformations, i.e., stabilization of T conformations, to the same extent as with SAM. This effect appears, however, at markedly higher ligand concentrations (25% AT at 25 µM SAH (Fig. 3c)), confirming the much lower affinity reported earlier by Winkler et al. [16]. They suggested that the binding pocket is sensitive to the absence of the single methyl group from the sulfur atom and the associated loss of positive charge (see Fig. 2a,b). In the aptamer domain of the *T. tengcongensis* SAM-I riboswitch, SAH forms the same set of hydrogen-bonding interactions as SAM and even places the neutral sulfur atom at the same position as the sulfonium ion of SAM [38]. Although SAH binds in the same manner as SAM, the lacking electrostatic interactions with the P1 helix have been made responsible for maintaining the riboswitch in a state that permits full transcription of the mRNA [38].

## Conformational dynamics

**Fig. 4:**
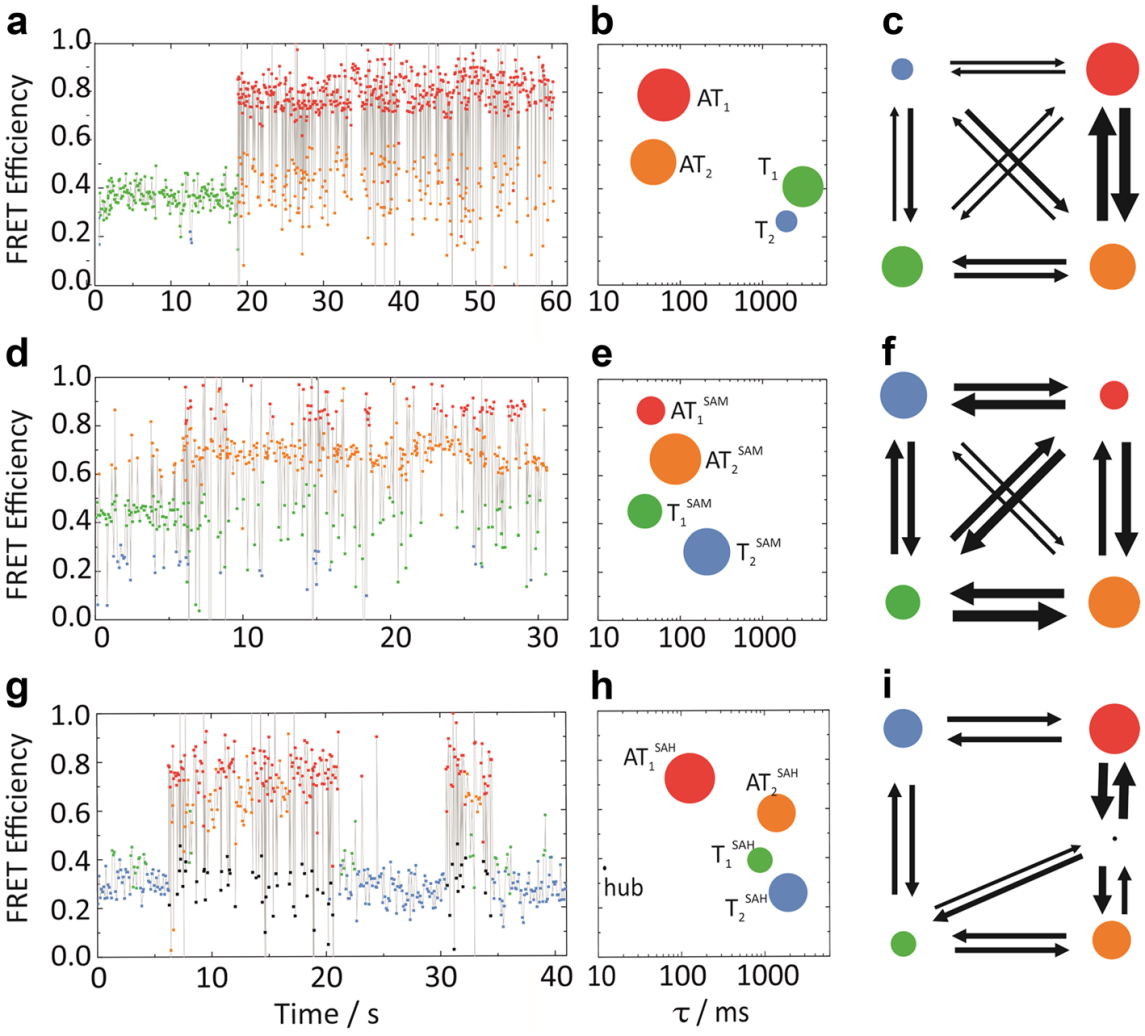
Hidden Markov model (HMM) analysis of SAM-I riboswitch conformational dynamics. **a** Typical smFRET efficiency time trace measured on an immobilized riboswitch exposed to buffer conditions with 15 mM Mg^2+^. Data were averaged in 100 ms time bins or less for shorter intervals. The assignment of the data points to different states by HMM analysis is indicated by different colors. **b** Two-dimensional scatter plot of the states identified by HMM, represented by disks with areas proportional to their equilibrium populations, as a function of FRET efficiency and lifetime. **c** Kinetic network of state interconversions. Different widths of the arrows represent fluxes (equilibrium population × rate coefficient) on the orders of 10^−3^ (thick)‒10^−5^ (thin) s^−1^. **d–f** Data corresponding to those in **a–c** for riboswitch molecules in buffer containing 15 mM Mg^2+^ and 10 μM SAM. **g–i** Data corresponding to those in **a–c** for riboswitch molecules in buffer containing 15 mM Mg^2+^ and 10 μM SAH

The FRET histograms (Fig. 3) have in common that the high-FRET and low-FRET distributions are very broad, indicating either continuous intrinsic heterogeneity or the presence of two or more unresolved distinct conformations. To characterize individual conformations and the interconversion dynamics between the states, we recorded emission intensity time traces in the green and red color channels of the riboswitch in buffer containing 15 mM Mg^2+^ in the absence of ligands, and with SAM and SAH added. At this particular Mg^2+^ concentration, roughly equal populations of molecules are present in AT and T conformations [30], ensuring a large number of interconversion events. From the data, we then calculated smFRET efficiency time traces. Selected FRET efficiency time traces in Fig. 4a, d and g show that the SAM-I riboswitch fluctuates between high-FRET and low-FRET states in the presence and absence of ligands. By using HMM analysis, two high-FRET states and two low-FRET states (for SAM, three for SAH) were resolved. In Fig. 4b, e and h, these conformations are represented by disks in a two-dimensional plot as a function of FRET efficiency and lifetime, with areas proportional to the equilibrium populations. The kinetic networks of state interconversion are plotted in Fig. 4c, f and i. Different thickness of the arrows encodes flux (equilibrium population times rate coefficient) between states, with thick lines representing high flux.

### Interconversion dynamics in the absence of metabolites

For the ligand-free riboswitch sample, HMM analysis revealed a structurally compact (high-FRET) pair of states, AT_1_ and AT_2_, with average lifetimes of a few ten milliseconds (Fig. 4b), as reported earlier [30]. Likewise, we identified a structurally extended (low-FRET) pair of states, T_1_ and T_2_, with long lifetimes of a few seconds (Fig. 4b). HMM analysis revealed interconversions between all four states. Figure 4c shows that the dynamics are dominated by fast (millisecond) switching between the two AT conformations, whereas transitions between the T conformations and between T and AT conformations are much less frequent and occur on timescales greater than seconds. Apparently, high free energy barriers and, presumably, major base pairing rearrangements are involved in these transitions.

In previous work [30], we used the HMM-derived information about the conformations and their FRET efficiencies to perform a global analysis of a set of FRET histograms for Mg^2+^ concentrations ranging from 0 to 100 mM. Four Gaussian distributions, depicted by colored lines in Fig. 3, with peak areas (fractional populations) and positions (average FRET efficiencies) varying with Mg^2+^ concentration according to Langmuir isotherms, *X*([Mg^2+^]) = *X*(0) + Δ*X*/(1 + [Mg^2+^]_0.5_/[Mg^2+^]), described all histograms very well, revealing a midpoint concentration, [Mg^2+^]_0.5_, for the T → AT transition in the millimolar range, in agreement with recent findings [37]. Lu et al. [14] reported earlier that, in the presence of Mg^2+^, the aptamer domain is a globally well-structured moiety pre-organized by tertiary interactions that is capable of ligand binding. This Mg^2+^-induced pre-organization of the aptamer domain is essential for assuming a conformation that can rapidly and selectively bind the SAM-I ligand shortly after transcription [37, 39].

### Interconversion dynamics in the presence of metabolites

In the presence of SAM or SAH ligands, a mechanistic model would require (at least) eight states, i.e., four ligand-bound states in addition to the four ligand-free states discussed above. Such an analysis is truly challenging and, therefore, we have limited ourselves to studying the dynamics at ligand concentrations much greater than the reported *K*_D_ values, to ensure that the ligand-bound fraction is maximized. We note, however, that this approach, guided by the ‘overall affinity’, does not completely ensure that the four conformational species are individually saturated with ligands. However, the fact that the results presented below show that the presence of SAM affects properties of all four conformations suggests that they are SAM-bound species.

The presence of SAM (10 µM, in buffer solution supplemented with 15 mM Mg^2+^) results in slightly increased FRET efficiencies of all four conformations (cmp. Figure 4b, e), which suggests a compaction due to the additional stabilization by the bound SAM. Moreover, SAM binding also affected the fractional populations of the four conformations (see Figs. 3b, 4e). Whereas AT_1_ and AT_2_ are the predominant conformations without SAM (Fig. 4b), AT_2_ and T_2_ are populated the most in the presence of SAM (Fig. 4e). These changes are only gradual, but a huge difference is seen in the interconversion dynamics, as is already evident from comparing the smFRET efficiency time traces (Fig. 4a,d). The lifetimes of the two AT (high-FRET) conformations are comparable to those in the absence of SAM; the two (low-FRET) T conformations, however, have lifetimes that are much shorter than in the absence of SAM. Transitions between AT_1_^SAM^ and AT_2_^SAM^ are somewhat less frequent (Fig. 4f). All other interconversions, however, are substantially accelerated upon SAM binding and appear on timescales of 10–100 ms, except for those between the AT_2_^SAM^ and T_2_^SAM^ states. Thus, SAM binding not only affects the fractional populations (i.e., the relative free energies) of the observed states, but also markedly changed the barriers separating them from one another in the conformational free energy landscape.

With 10 µM SAH added to the buffer solution, HMM again resolved two high-FRET AT and two low-FRET T states. In addition, a third low-FRET state with a small fractional population and a very short lifetime of only 12 ms was found. This state is visible via the short and frequent excursions to lower FRET values in the FRET efficiency traces in Fig. 4g (black points) and denoted as ‘hub’ in Fig. 4h. This result illustrates the power of HMM, showing that the hub state is well distinguishable from T_1_ and T_2_ by its short lifetime, although its FRET efficiency distribution completely overlaps with these states. The relative populations of the four major conformations were different from those without and with SAM. The high-FRET state AT_1_ still had a short state lifetime, about two-fold greater than in the absence of a ligand (Fig. 4h). However, the lifetime of the high-FRET state AT_2_ was more than an order of magnitude greater than in the presence and absence of the SAM ligand. The low-FRET states T_1_ and T_2_ in the presence of SAH are long-lived, similar to those without a ligand present. The kinetic network indicates that the hub state is visited during the exchange between the AT_1_ and AT_2_ states and for transitions to T_1_ (Fig. 4i). Its short lifetime of 12 ms and its lower average FRET efficiency than that of AT_1_ and AT_2_, suggests that this ‘transition state’ has maintained its secondary structure but has a modified tertiary fold with a greater inter-dye distance. Based on these findings and its connectivity en route between AT_1_ and AT_2_ (Fig. 4i), we tend to assign it to an AT state. Interestingly, HMM analysis did not resolve any direct transitions between the AT_1_ and T_1_ and AT_2_ and T_2_ states. In addition, comparison of the dynamics between SAM and SAH (Fig. 4f, i) reveals that the pronounced acceleration of conformational interconversions between AT and T states observed upon SAM binding is not present for SAH. In summary, the riboswitch is significantly less dynamic with SAH than with SAM bound.

## Discussion and conclusions

The energy landscape has proven to be an extremely powerful concept to analyze protein folding and function [5, 40–42]. In recent years, a hierarchical energy landscape model with multiple tiers (0, 1 and 2) has been adopted for RNA molecules, much in the vein of Hans Frauenfelder’s depiction of the energy landscape of carbonmonoxy myoglobin [12]. Here we have explored the structure and dynamics of an RNA energy landscape using smFRET spectroscopy. The strong distance dependence of the FRET coupling allowed us to distinguish different states in the conformational energy landscape. By measuring the fluorescence emission and analyzing the FRET efficiency of a donor–acceptor dye pair specifically attached to the RNA, we collected FRET histograms, distributions of FRET efficiencies characterizing the ensemble. For small RNA molecules such as tRNA, ribozymes or riboswitches, these histograms are typically broad and can be decomposed into contributions from distinct conformations (Tier 0 states), which is often done by fitting multiple Gaussians [29, 35] (Fig. 3). Oftentimes, this separation is ambiguous because of strong overlap. However, the equilibrium populations (fractional areas) are usually found to vary with the concentration of ligands or counterions, most importantly, Mg^2+^, in specific ways. Exploiting this dependence can greatly aid in unraveling overlapping contributions by multiple Tier 0 conformations, and one may also gain insight into the nature of these conformations. In addition to identifying discrete FRET peaks, these peaks are also seen to shift continuously with changing counterion concentration, indicating structural rearrangements in the lower tiers of the energy landscape. In contrast to steady-state experiments, time-resolved smFRET experiments on immobilized RNA molecules are much more tedious to carry out. They can greatly assist in the identification of discrete conformations, however, as both spectroscopic and temporal information are used in combination within HMM data analysis (Fig. 4) [34]. Moreover, the kinetics of conformational transitions becomes directly accessible to measurement. Still, a word of caution is in order concerning the information gained from smFRET. Projecting the multi-dimensional energy landscape onto a single FRET coordinate is an utter simplification and can provide only limited insight. In fact, attaching the FRET donor and acceptor dyes at different positions may lead to different results; e.g., two conformations distinguishable with a certain FRET pair may completely overlap when using another one [43].

When we started our smFRET investigations of the SAM-I riboswitch, we anticipated to find a simple energy landscape with two states, AT and T, displaying marked changes in their fractional populations (free energies) upon metabolite binding and thereby communicating the SAM concentration to the RNA polymerase (RNAP)-mediated transcription process. Instead, we found four distinct conformations, which were assigned to two T and two AT conformations, which were appreciably populated under all conditions (except at very low Mg^2+^ concentration). To our surprise, there were only modest shifts of the fractional populations toward low-FRET T states upon addition of ligands (see Fig. 3), as has also been reported for other riboswitches [44]. This result raises the question as to how the riboswitch can exert its biological function. The data shown here suggest that equilibrium properties are not important for the function, but rather the dynamics of conformational transitions, which is governed by the barriers between the states, as reported earlier for other riboswitches [44–46].

To appreciate the role of dynamics in the biological function of the SAM-I riboswitch, we note that this RNA molecule, as a transcriptional riboswitch, senses the presence of the ligand while the RNAP synthesizes the mRNA strand encoding the riboswitch and, further downstream, the enzyme to be controlled. Thus, there is only a brief window of opportunity after the RNAP has transcribed the riboswitch that transcription can be terminated. This interval is determined by the processivity of the RNAP (~ 40–90 nt s^‒1^ according to Ref. [47]) and, possibly, pausing sites on the mRNA. During this time, the aptamer domain has to fold quickly so that it becomes competent to bind SAM in a bimolecular reaction, the rate of which scales with the SAM concentration. In addition, conformational changes have to occur so that the population of T conformations is enhanced to suppress continued transcription.

Indeed, the aptamer domain spontaneously folds into a compact, well-structured domain at physiological Mg^2+^ concentrations ready to bind a ligand, as reported in many papers [15, 24, 30, 37, 39, 48, 49]. The nascent riboswitch is known to have a propensity for assuming an AT conformation [25, 50, 51]. Therefore, in the absence of SAM, a large fraction of riboswitch molecules remain in the transcriptional ON state (AT conformation), typically for several seconds, as transitions between AT and T states are slow without SAM bound. If SAM binds at an early stage, however, even though the AT conformation is initially formed, the dramatic acceleration of interconversion between AT and T states (Fig. 4e–f) that we observed may enable the riboswitch to swiftly convert to the thermodynamically more stable T conformation, i.e., the transcriptional OFF state. Notably, our findings with SAH are also in line with this scenario. First, SAH binds to the SAM-I riboswitch with a much lower affinity. It shows a similar, weak population shift toward T conformations as SAM (but at much higher ligand concentrations, Fig. 3), but its accelerating effect on the dynamics is much weaker (cmp. Figure 4c, f, i). Consequently, SAH is expected to be less efficient in inducing terminating riboswitch states.

To conclude, our smFRET experiments suggest that the accelerated conformational dynamics upon SAM binding is crucial for the proper biological function of the SAM-I riboswitch. While this scenario appears entirely reasonable, we note that our experiments do not probe the riboswitch in the real biological situation, i.e., while the RNAP processes the mRNA strand. Therefore, we anticipate further surprising results when measuring conformational dynamics of the riboswitch during transcription.
